# Detection of sub-nmol amounts of the antiviral drug favipiravir in ^19^F MRI using photo-chemically induced dynamic nuclear polarization

**DOI:** 10.1038/s41598-024-51454-4

**Published:** 2024-01-17

**Authors:** J. Bernarding, C. Bruns, I. Prediger, M. Mützel, M. Plaumann

**Affiliations:** 1 Institute of Biometry and Medical Informatics, Otto-von-Guericke University Magdeburg, Leipziger Strasse 44, 39120 Magdeburg, Germany; 2 Pure Devices GmbH, 97222 Rimpar, Germany

**Keywords:** Biomarkers, Medical research, Chemistry

## Abstract

In biological tissues, ^19^F magnetic resonance (MR) enables the non-invasive, background-free detection of ^19^F-containing biomarkers. However, the signal-to-noise ratio (SNR) is usually low because biomarkers are typically present at low concentrations. Measurements at low magnetic fields further reduce the SNR. In a proof-of-principal study we applied LED-based photo-chemically induced dynamic nuclear polarization (photo-CIDNP) to amplify the ^19^F signal at 0.6 T. For the first time, ^19^F MR imaging (MRI) and spectroscopy (MRS) of a fully biocompatible model system containing the antiviral drug favipiravir has been successfully performed. This fluorinated drug has been used to treat Ebola and COVID-19. Since the partially cyclic reaction scheme for photo-CIDNP allows for multiple data acquisitions, averaging further improved the SNR. The mean signal gain factor for ^19^F has been estimated to be in the order of 10^3^. An in-plane resolution of 0.39 × 0.39 mm^2^ enabled the analysis of spatially varying degrees of hyperpolarization. The minimal detectable amount of favipiravir per voxel was estimated to about 500 pmol. The results show that ^19^F photo-CIDNP is a promising method for the non-invasive detection of suitable ^19^F-containing drugs and other compounds with very low levels of the substance.

## Introduction

**Figure 1:**
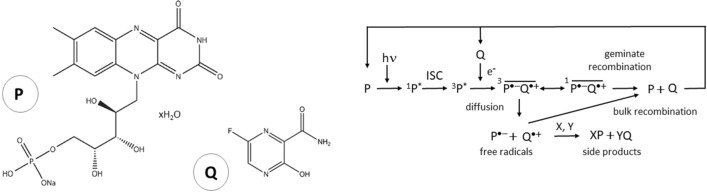
Molecular structures and spin-selective chemical reaction pathways in liquid-state photo-CIDNP (modified after^16^). *Left image*: Molecular structures of riboflavin-5′-monophosphate (chromophore P) and of the fluorinated antiviral drug (hyperpolarizable target molecule Q). *Right image:* Simplified reaction pathways in liquid-state photo-CIDNP. Absorption of light (hν) generates an excited singlet state in P, which subsequently transitions via intersystem crossing (ISC) into a highly reactive molecular triplet state. This triplet attracts an electron from the donor Q thereby forming a spin-correlated radical pair (SCRP) with conserved electron spin symmetry. Due to the different Larmor frequencies of the two electrons, the SCRP oscillates between a triplet and a singlet state^31^. Hyperfine interactions and the difference of the Landé factors (Δg) of the electrons of the radical pair lead to spin sorting of the nuclear spin states which finally gives rise to a non-Boltzmann enrichment of the nuclear spins. The geminate recombination into the original educts can only take place from the singlet state of the SCRP. In the second pathway, the molecules split into free radicals (escape products) and diffuse apart from the solvent cage into the surrounding solvent*.* There, they recombine either into the original educts (bulk recombination) or they may form side products with other molecules X, Y. For more details see^14,17,32^.

Favipiravir (6-fluoro-3-hydroxypyrazine-2-carboxamide) belongs to the class of fluorinated pyrazine carboxamides (Fig. 1) and is the main component of an antiviral drug that has been used in some countries for oral treatment of various RNA virus infections^1^, including COVID-19 and Ebola.

^19^F, which is found in many pharmaceuticals, is of particular interest since no natural ^19^F is present in the human organism. Therefore, ^19^F provides a background-free marker for NMR and MRI^2^. However, the concentration of the corresponding marker molecules is often very low, and MR methods are quite insensitive compared to other molecular imaging techniques. The overarching goal of our project is therefore to research and establish new approaches to detect even very small amounts of ^19^F-containing biomarkers.

The main strategy to increase the SNR is applying higher magnetic fields (B_0_ field) to increase the so-called thermal (Boltzmann) polarization. The SNR is approximately a quadratic function of the magnetic field strength^3^ although other factors such as coil characteristics also play an important role. For clinical applications 7 T whole body MRI was recently introduced, and even higher magnetic fields are already available for human research MRI and experimental MRS^4^. However, in addition to being very expensive, ultra high field MRI also poses unique technical problems and altered relaxation times, all of which affect image contrast.

A completely different approach is to increase the spin population difference beyond the thermal Boltzmann distribution by hyperpolarization techniques, which can provide up to several orders of magnitude higher SNR^5^. There exist different hyperpolarization techniques such as *dynamic nuclear polarization* (DNP)^5^, *spin exchange by optical pumping* (SEOP)^6^, or para-hydrogen-based techniques such as standard *para-hydrogen-induced polarization* (PHIP)^7^ and *signal amplification by reversible exchange* (SABRE)^8^.

Interest in these techniques for biomedical applications was sparked by the demonstration that MRI signals could be significantly enhanced by hyperpolarization, allowing MRI- and MRS-based detection of the metabolism of tumor-related molecules such as pyruvate^5,9,10^. However, they typically require the removal of toxic substances involved in the hyperpolarization generation process, or complex, costly engineering facilities to thaw the substances hyperpolarized at just a few Kelvin and inject them within the hyperpolarization decay time (DNP). In addition, they require sophisticated experimental modifications if being used repetitively.

An alternative hyperpolarization technique is based on special spin-selective chemical reactions (*chemically induced dynamic nuclear polarization*, CIDNP), in which a spin-correlated electron radical pair is generated, which ultimately leads to a non-Boltzmann polarization of the target nucleus (Fig. 1). CIDNP was discovered 1967 independently by two groups^11–13^. If the initial radical is generated by irradiating a photo-sensitive molecule that builds a radical pair with the target molecule the technique is called photo-CIDNP. Photo-CIDNP can be used not only to hyperpolarize ^1^H^14^ but also ^19^F^15–17^ or other nuclei such as ^13^C^18^ or ^15^N^19–21^. As an advantage over other hyperpolarization methods such as DNP or PHIP/SABRE, photo-CIDNP can be performed in biologically compatible solutions. It is also a partially cyclic reaction, allowing many repetitions until the photo-sensitive chromophore is bleached (Fig. 1). Repeating measurements can serve to increase the SNR by signal averaging as well as to monitor the dynamic evolution of the system^22^. Replacing the potentially harmful lasers with high-power LEDs to excite the molecule not only reduces the cost of the experimental setup, but also makes it extremely safe to operate in normal laboratory or medical settings^23,24^. Photo-CIDNP has long been applied to biomedical important model systems such as amino-acids and ^19^F-labeled proteins^17,25–27^. The magnetic field dependent spin dynamics of light-induced radical pairs in retinal cryptochrome flavoproteins could even explain the magnetic sense of some migratory birds^28^.

So far, however, only a few spatially resolved measurements based on photo-CIDNP have been reported^15,16,29,30^. Recently we showed that at 0.6 T the field-dependent CIDNP-effect is strong enough and the lifetime of the hyperpolarization is sufficiently long to allow performing ^19^F MRI. In that study, we investigated a biocompatible aqueous solution containing 3-fluoro-tyrosine and riboflavin-5′-monophosphate as a chromophore^16^.

In this proof-of-principle study we investigated a fully biocompatible model of an aqueous solution of the fluorinated antiviral drug favipiravir as the target molecule with riboflavin-5ʹ-monophosphate (FMN) as a chromophore (Fig. 1). As in a previous study, the experimental setup not only allows ^19^F and ^1^H MRI but also NMR-spectroscopic measurements including the simultaneous data acquisition of both ^1^H and ^19^F. Further technical details on the experimental setup can be found in^16^ and in the discussion section below.

## Results

### ^1^H and ^19^F MRI

**Figure 2:**
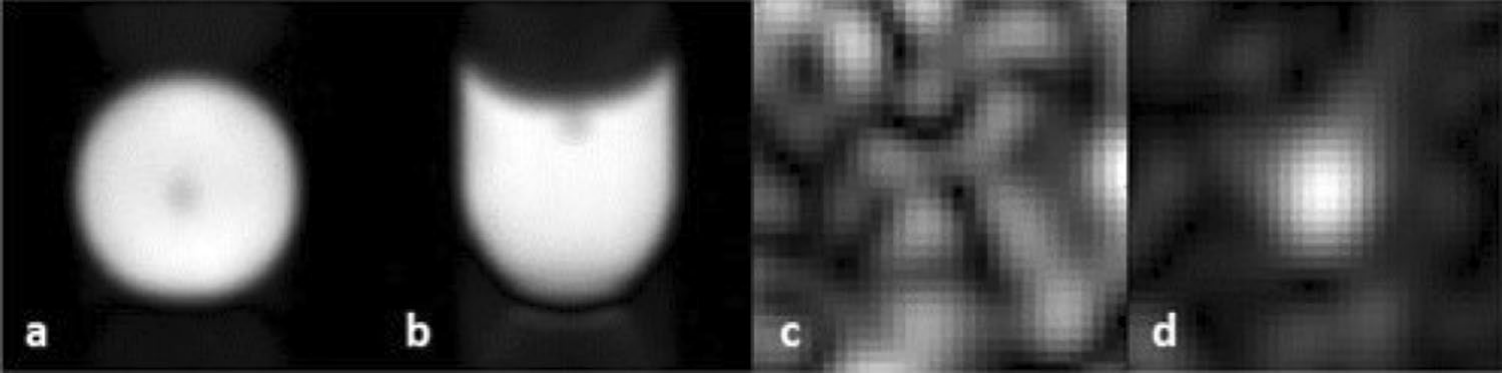
Two-dimensional Turbo spin echo (2D-TSE) ^1^H and ^19^F MRI. (**a**, **b**) High-resolution ^1^H images of the sample (one average, matrix size 32 × 32, TE 6 ms, phase-oversampling (PhOS) factor 8, read-oversampling factor 2, field-of-view (FoV) 15 × 15 mm^2^, one slice covering the whole sample, data fourfold zero-filled). The central signal decrease reflects the position of the fiber. Due to the two-dimensional acquisition technique, the image represents a projection along the direction orthogonal to the displayed plane. **a**: ^1^H image, axial view, **b**: ^1^H image, coronal view. (**c**, **d**) ^19^F images without (**c**) and after 4 s of illumination (**d**) (one average, axial view, matrix size 8 × 8, FoV 15 × 15 mm^2^, one slice covering the whole sample, PhOS 4, Turbo factor (TF) 32, zero-filling factor 4). Without illumination, only noise can be seen, while after illumination the signal is clearly visible in the center of the image.

The capability to observe ^1^H and ^19^F signals simultaneously allowed a quick and reliable adjustment of the sequence parameters and illumination power to optimize data acquisition both for spectroscopy and MRI (see methods section and Fig. 2). The ^1^H images (Fig. 2a and b) were used to verify fiber and sample positioning. They were acquired with a higher resolution than the ^19^F images. The excellent spatial resolution is evident from the clear delineation of the fiber tip within the sample, even though the ^19^F coil exhibits only 15% sensitivity at the ^1^H Larmor frequency compared to the ^19^F frequency.

No signal was seen in ^19^F MRI when the sample was not illuminated (Fig. 2c). This changes dramatically after illumination of the sample. A spatially localized signal is now clearly visible (Fig. 2d). We started with a low-resolution image as the approximate spatial position and strength signal can be estimated after just one measurement. The one-shot measurement also minimizes potential light-induced damage to the chromophore.

**Figure 3:**
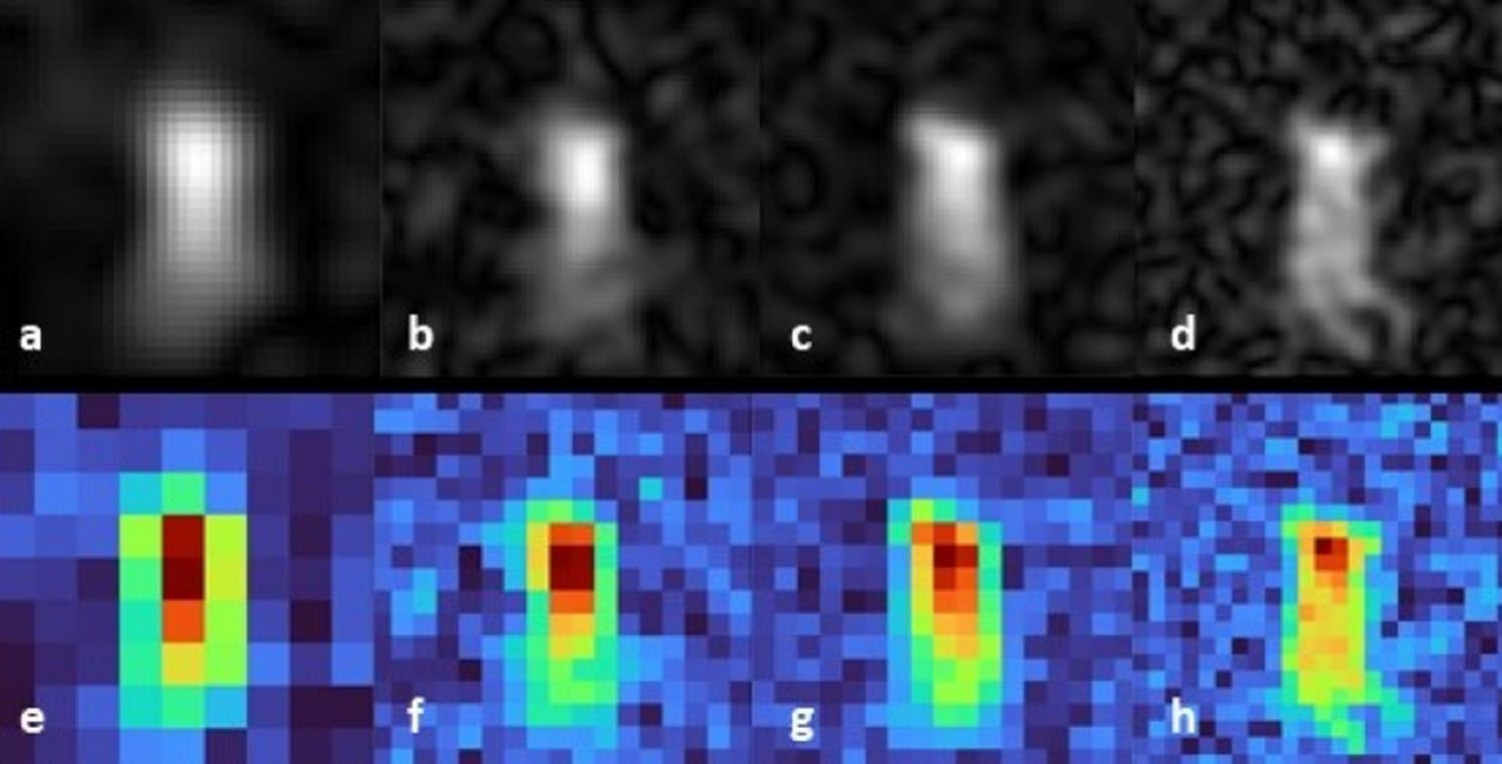
Examples of different spatial distributions of ^19^F hyperpolarization of favipiravir with varying resolution and number of acquired k-space lines (Coronal 2D-TSE, FoV 9 × 9 mm^2^, slice thickness 9 mm, PhOS 4, TE 4 ms, TR 4 s). (**a**–**d**) gray value images (zero-filling factor 4). (**a**) matrix 9 × 9, 8 averages, TF 36; (**b**) matrix 17 × 17, 16 averages, TF 68, PhOS 4; (**c**) matrix size 17 × 17, 16 averages, TF 17; (**d**) matrix size 23 × 23, 16 averages, TF 46. (**e**–**h**) Corresponding non-zero-filled images showing the varying degree of hyperpolarization in original resolution. The colors correspond to the measured values in μV displayed in each image (red/blue: highest/lowest signal, cf. Fig. 6). Figure 3b/f and c/g have identical resolution but Fig. 3b/f was acquired using one measurement cycle covering the whole k-space while figure c/g was acquired using four cycles each covering a quarter of the four-fold oversampled k-space. Figure 3d/h was acquired using two acquisition cycles (acquisition of one cycle required a new illumination; see text for details).

In the next step, we increased the resolution by reducing the field of view and increasing the image matrix (Fig. 3; the matrix size was changed to odd numbers to allow the positioning of the fiber tip in the center of the sample). Images were oversampled in read-out and in phase-encoding directions, the latter increasing the acquisition time by the factor of phase-encoding oversampling which for short *T*_*2*_-values may lead to signal loss in the acquisition of late echoes (see discussion section). Increasing the resolution resulted in more detail, but also increased the noise, if the number of averages is increased (see Table 1).

**Table 1:** Image acquisition parameters, measured values of noise as well as the signal and SNR of the totally illuminated area (*S*_*total*_), and the segmented high intensity (*S*_*high*_) and low intensity parts (*S*_*low*_) (cf. Figure 3). The fourth row presents the time required for acquiring the number of k-space lines after one block of illumination. All images were acquired with a FoV of 9 × 9 mm^2^ (cf. Figure 3) and a four-fold oversampling, i.e. four-fold number of k-space lines as compared to the actual resolution. The first two echoes of each echo train were not used for image encoding to reduce the impact of B_0_ and B_1_ artifacts.

Figure	3a/e	3b/f	3c/g	3d/h
Image acquisition parameters				
Actual resolution in plane (FoV 9 × 9 mm^2^)	9 × 9 1 mm^2^/pix	17 × 17 (0.53)^2^ mm^2^/pix	17 × 17 (0.53)^2^ mm^2^/pix	23 × 23 (0.39)^2^ mm^2^/pix
Acquisition (per illumination)	Full image (36 k-space lines)	Full image (68 k-space lines)	Full image/4 (17 k-space lines)	Full image/2 (46 k-space lines)
Illumination	1 × 4 s	1 × 4 s	4 × 4 s	2 × 4 s
Acquisiton time per illumination	36 × 4 ms + 8 ms = 152 ms	68 × 4 ms + 8 ms = 280 ms	17 × 4 ms + 8 ms = 76 ms	46 × 4 ms + 8 ms = 192 ms
Averages	8	16	16	16
Signal-to-noise ratio (SNR)				
Noise/μV	2.73 × 10^–4^	4.27 × 10^–4^	5.0 × 10^–4^	6.5 × 10^–4^
Mean S_total_/μV SNR_total_	1.68 × 10^–3^ 6.15	2.02 × 10^–3^ 4.73	3.07 × 10^–3^ 6.14	2.77 × 10^–3^ 4.26
Mean S_high_/μV SNR_high_	3.01 × 10^–3^ 11.03	3.78 × 10^–3^ 8.85	5.05 × 10^–3^ 10.1	4.68 × 10^–3^ 7.2
Mean S_low_/μV SNR_low_	1.48 × 10^–3^ 5.42	1.79 × 10^–3^ 4.19	2.99 × 10^–3^ 5.98	2.65 × 10^–3^ 4.0
Signal_min_/μV (blue) Signal_max_/μV (red)	6.39 × 10^–5^ 3.17 × 10^–3^	1.71 × 10^–5^ 3.96 × 10^–3^	2.04 × 10^–5^ 5.481 × 10^–3^	6.14 × 10^–5^ 5.24 × 10^–3^

We then applied the segmented TSE method by acquiring multiple shorter echo trains (here: four echo trains) to test whether finer details could be displayed with a higher amplitude. Figure 3c shows more details, however, not only the 90° pulse had to be repeated four times but also the preceding irradiation. The SNR of the single images was too low to show the ^19^F signal unambiguously, but averaging led to a clear depiction of the illuminated area. As routinely used in clinical diagnosis the grey-valued images in Fig. 3a–d are zero-filled resulting in a finer resolution. The original image resolution is shown in Fig. 3e–h, whith color-coding supporting the visual inspection of the spatial distribution of the hyperpolarization.

Compared to our previous study on ^19^F tyrosine^16^, we did not acquire images of non-illuminated favipiravir as this would have required also a long acquisition time similar to the long-term acquisition of non-illuminated spectra, where the substance showed signs of structural changes after being in the magnet for many hours (see discussion section and SI).

Acquisition parameters, mean signals and SNR of the different parts of the illuminated area for data of Fig. 3 are summarized in Table 1. Values were determined using a histogram-based segmentation procedure (see Fig. 6). While the absolute signals were comparable between Fig. 3c/g and d/h for both the central part *I*_*1*_ with the strongest hyperpolarization and the peripheral part *I*_*2*_, the background noise is higher at increased resolution. The influence of signal loss due to *T*_*2*_ was reduced in Fig. 3c/g as the time for acquiring a part of k-space after illumination was shorter than in the other images. This was due to the four-fold segmentation of the image acquisition and the according four-fold illumination of the sample. For further effects to be considered see discussion section. Thus, Fig. 3c/g exhibited a high SNR at medium-high resolution.

### ^19^F MR spectroscopy, relaxation times and estimation of signal enhancement

**Figure 4:**
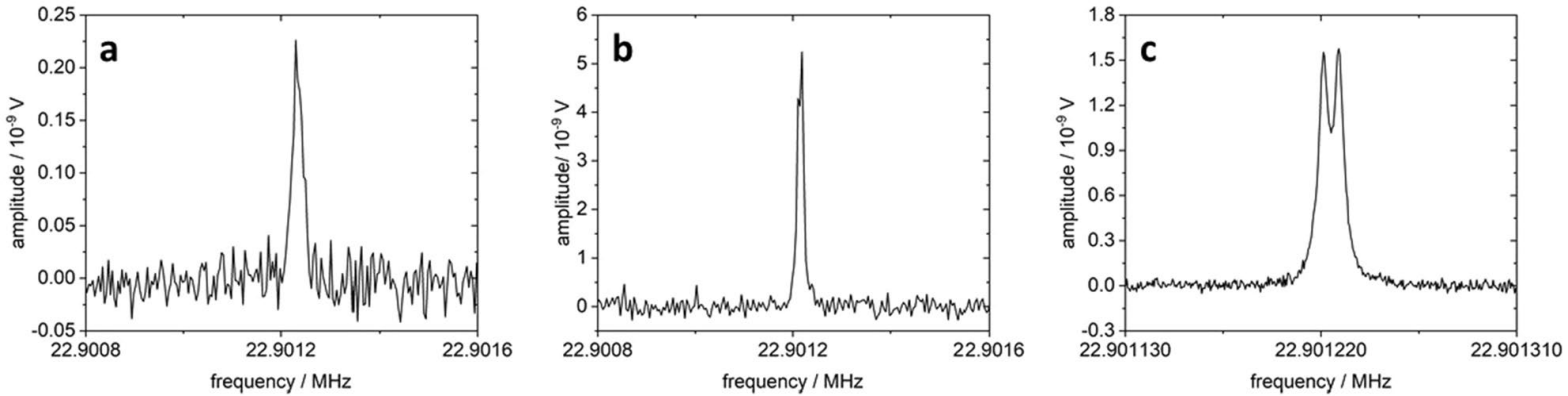
LED-induced signal enhancement of favipiravir in H_2_O (phase-corrected real part of spectrum). (**a**) Non-hyperpolarized favipiravir (2048 averages, TR 10 s, FID acquisition time 250 ms); (**b**) Hyperpolarized favipiravir (4 s illumination, 32 averages, TR 10 s, FID acquisition time 250 ms). (**c**) High-resolution spectrum of hyperpolarized favipiravir (128 averages, 4 s illumination, TR 10 s, FID acquisition time 2 s).

Spectroscopic measurements served to estimate the initial hyperpolarization of the sample, the relaxation times, and the signal enhancement (SE). As a first estimation of the degree of hyperpolarization, we used the standard procedure of acquiring spectra with and without illumination. Typical measurements are shown in Fig. 4a and b. Without illumination, several thousand acquisitions were required (measurement time 341 min) to achieve an acceptable SNR, while hyperpolarized spectra required only one to a few data acquisitions depending on the spectral resolution (measurement time 5.3 min in Fig. 4b). Thus, hyperpolarization resulted in this example in a 64-fold reduction in data acquisition time while simultaneously increasing the signal amplitude by approximately 20-fold.

Because the low-resolution hyperpolarized spectra showed evidence that the ^19^F resonance line might have an additional substructure, we acquired a higher-resolution spectrum (Fig. 4c). The clearly resolvable doublet is due to J-coupling between the ^19^F and the neighboring ^1^H nucleus with J = 8.4 Hz. However, due to the longer acquisition time, the resulting increased noise required significantly more averaging to achieve a good SNR. This acquisition scheme is therefore not suitable for acquiring a large number of spectroscopic measurements due to increasing potential bleaching effects.

**Figure 5:**
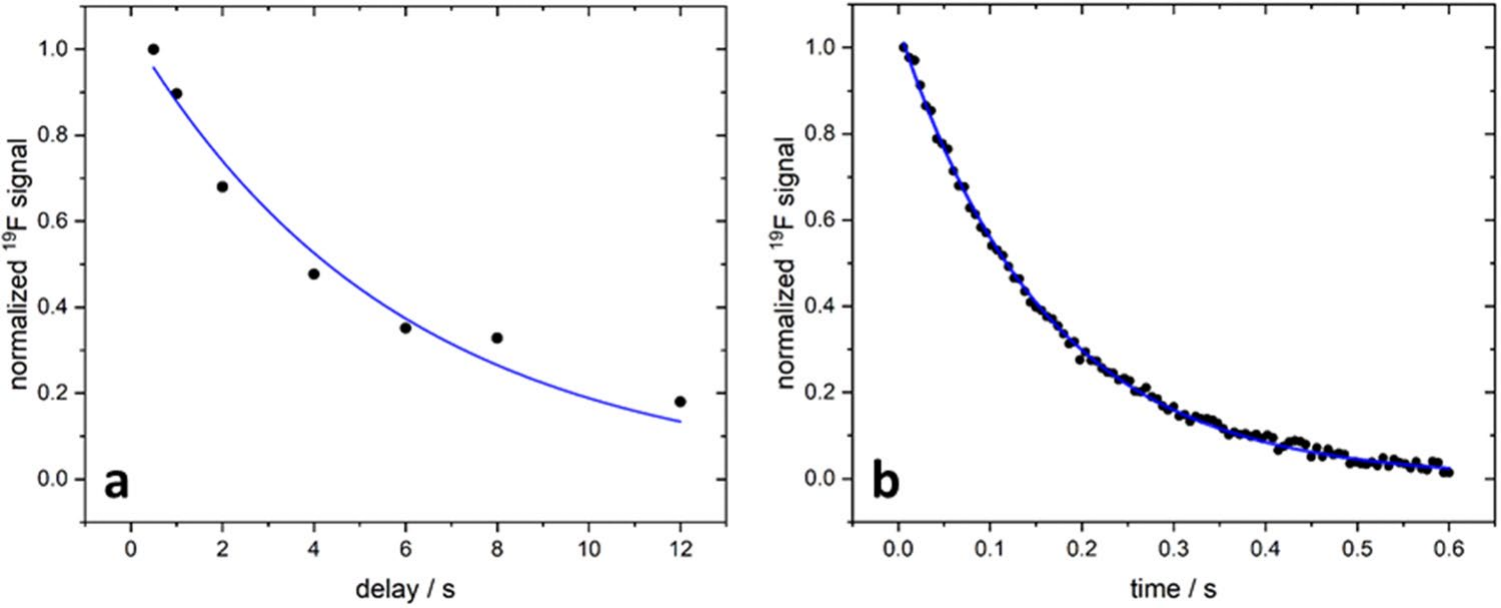
Relaxation times of ^19^F in favipiravir. (**a**) Lifetime (*T*_*1*_-relaxation) of hyperpolarized ^19^F as a function of the delay after illuminating the sample for 6 s (repetition time TR kept constant at 20 s). Each spectroscopic data set was acquired four times and then averaged except for two longest delay times (eight acquisitions to increase the SNR by averaging). The blue line depicts the mono-exponential fit with a half lifetime of (5.8 ± 0.6) s. (**b**) *T*_*2*_-relaxation measured using a CPMG sequence and acquiring 100 echoes with TE = 6 ms, TR = 20 s (illumination time 4 s). 32 data sets were averaged. *T*_*2*_ was determined with a mono-exponential fit to (159 ± 8.8) ms (blue line).

We then determined the *T*_*1*_-relaxation time from the decay of the hyperpolarization as a function of the delay between illumination and acquiring the FID leading to a *T*_*1*_ of about 6 s (Fig. 5a). The transversal lifetime of the hyperpolarization was determined using a Carr-Purcell-Meibohm-Gill (CPMG) pulse sequence leading to a *T*_*2*_-relaxation time of about 160 ms (Fig. 5b). The *T*_2_* times of ^1^H and and hyperpolarized ^19^F were in the range of 32 ms (Fig. SI 2).

**Figure 6:**
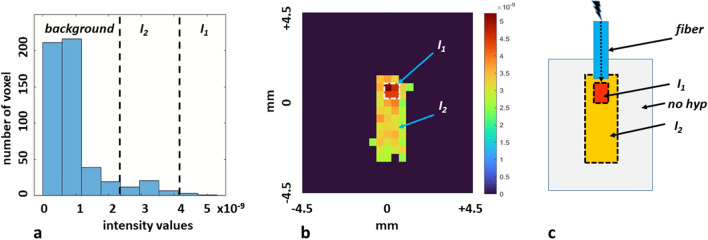
Spatial distribution of the ^19^F hyperpolarization of favipiravir (compare Fig. 3d and h). (**a**) Histogram of the intensities color-encoded in Fig. 3h, 6b (dashed lines symbolize cut-offs of image values). (**b**) Threshold image (compare Fig. 3h) to determine the spatial distribution of the degree of hyperpolarization (colors encode for the signal in V). (**c**) Schematic view of the fiber, the volume *V*_*1*_ with high mean intensity *I*_*1*_ and the volume *V*_*2*_ with low mean intensity *I*_*2*_.

To determine the SE of ^19^F, it has to be taken into account that the sample is inhomogeneously illuminated preventing a simple comparison of the spectra with and without illumination: the signal of the non-illuminated spectrum originates from the entire volume, while the signal after illumination comes from a much smaller volume. Therefore, we included image-based information to estimate the mean SE. The resolution of the digitized image makes it difficult to precisely analyze the distribution of the hyperpolarized volume especially in the border zones exhibiting partial volume effects. In a first rather crude approximation, we estimated two partial volumes *V*_*1,2*_ exhibiting a clearly delineated high and low mean degree of hyperpolarization. The extensions of the hyperpolarized regions were determined using a histogram-based approach (Fig. 6a and b) by manually adjusting the thresholds for the intensities to exclude noisy as well as partial volume voxels resulting in a somewhat higher mean intensity for the peripheral parts. *I*_*1*_ and *I*_*2*_ were then determined by averaging the values in each partial volume. Although the 2D imaging technique results in a rectangular volume where the intensities have been projected onto the image plane, the signals come only from smaller volumes that we assumed to be rotationally symmetric. The spatial distribution *ρ(x,y,z)* of the hyperpolarization can then be approximated as follows, where heights (*h*_*1*_, *h*_*2*_) and diameters (*d*_*1*_, *d*_*2*_) of the cylinders were determined from the transversal plane (Fig. 6c):

1 $$\rho \left( {x,y,z} \right) = \left\{ {\begin{array}{*{20}l} {I_{1} ,} \hfill & {\quad x_{1} ,y_{1} ,z_{1} \in V_{1 } } \hfill \\ {I_{2} ,} \hfill & {\quad x_{2} ,y_{2} ,z_{2} \in V_{2 } } \hfill \\ 0 \hfill & {\quad elsewhere} \hfill \\ \end{array} } \right.,\quad V_{1} = { }h_{1} \left( {\frac{{d_{1} }}{2}} \right)^{2} ,\quad V_{2} = { }h_{2} \left( {\frac{{d_{2} }}{2}} \right)^{2} - V_{1}$$

In the selected image, each quadratic pixel had a size of (0.39 mm)^2^, i.e., FoV/matrix size leading to *d*_*1*_ = 0.78 mm, *h*_*1*_ = 0.78 mm, and *d*_*2*_ = 1.56 mm, *h*_*2*_ = 4.29 mm. Making the assumption that the illumination from the cylindrical fiber has rotational symmetry, the resulting cylindrical volumes were then approximately *V*_*1*_ = 0.37 μL and *V*_*2*_ = (8.2–0.37) μL = 7.83 μL. The used phase encoding scheme (center-out) leads to a reduced amplitude at higher phase encoding steps (*T*_*2*_ effect). This leads to a similar effect to k-space filters that are typically used to reduce Gibbs ringing. Because of this, the apparent illuminated region detected in the images might be larger in phase encoding direction than the actually illuminated region.

The mean intensity *I*_*2*_ was determined to approximately 70% of the mean intensity *I*_*1*_. We assumed that each signal (hyperpolarized and non-hyperpolarized) is proportional to the number of hyperpolarized (*N*_*hyp*_) and non-hyperpolarized (*N*_*total*_) favipiravir molecules, which in turn is proportional to the volumes of the corresponding regions. The number of hyperpolarized molecules *N*_*hyp*_ was therefore estimated (where *α* denotes an unknown constant of proportionality) to

2 $$N_{hyp} = \alpha\left( {V_{1} + 0.{7} \cdot V_{2} } \right)$$

To determinate the mean SE factor, the signal of the non-illuminated sample which results from a much larger volume of 600 μL must therefore be scaled down by a correction factor that considers the smaller illuminated volume:

3 $$F_{CORR} = N_{{hy{\text{p}}}} {/}N_{total} = \left( {V_{1} + \, 0.{7}V_{2} } \right)/V_{{{\text{total}}}} = {5}.{85/6}00 = 0.00{98}$$

The phase-corrected real part of the ^19^F spectra with (*I*_*LED_ON*_) and without illumination (*I*_*LED_OFF*_) (Fig. 4a, b) was integrated over a range of 60–75 Hz (where the signals approached the baseline).

This led to *I*_*LED _ON*_ = 89.72·10^–9^ VHz and *I*_*LED _OFF*_ = 5.487·10^–9^ VHz. The mean SE was estimated considering the correction factor:

4 $${\text{SE}} = I_{LED\_ON} /I_{LED\_OFF} /F_{CORR} = {16}.{37}/0.00{98} = {167}0.$$

To estimate the minimum amount of hyperpolarized substance seen in Fig. 6b, we assumed that the most peripheral parts of the cylindrical volume *V*_*2*_ represent the projection of 2–3 cubic voxels. With a volume of 0.06 μL per voxel, this would correspond to less than 331–496 pmol of hyperpolarized favipiravir.

## Discussion

Several main results were obtained in this study: first, it was shown that the background-free ^19^F nucleus of the drug favipiravir, already used for anti-viral treatment in humans, could be hyperpolarized using an easy-to-apply and inexpensive experimental setup. Photo-CIDNP allowed to combine the light-induced signal amplification with standard NMR signal averaging on the same sample without adding new substances, and thus increasing the SNR as compared to single image acquisition. Second, the photo-CIDNP effect was large enough at 0.6 T to allow ^19^F MRI of favipiravir with a sub-mm resolution. Additionally, T_1_ and T_2_ relaxation times of hyperpolarized ^19^F in favipiravir could be determined. Third, the minimal amount of hyperpolrazed ^19^F detectable on MRI was about 500 pmol or less per voxel. Taken together, these results provide a major step towards significantly increasing the sensitivity of MRI and making it more applicable for molecular imaging techniques. Favipiravir is a very interesting molecule as it contains a pyrazine moiety. It can therefore also be hyperpolarized using para-hydrogen-based techniques. ^1^H hyperpolarization of favipiravir was recently reported by Jeong et al.^33^. In contrast to our approach, they used SABRE to hyperpolarize Favipiravir. However, they did not investigate a transfer of the ^1^H hyperpolarization to the ^19^F nucleus. Additionally, SABRE requires dissolving the drug in an organic solvent together with an Iridium-based catalyst, both of which are toxic. Therefore, this concept cannot be easily transferred to living organisms, as it would require rapid and complete removal of these substances before being applied. However, both techniques might be used in a complementary manner to analyze the different molecular mechanisms underlying generation and transfer of hyperpolarization between different nuclei.

It is important to note that the CIDNP effect depends not only on the substances used but also on the applied magnetic field. ^1^H photo-CIDNP of amino acids and other substances was analyzed over a large range of near-earth magnetic field up to 7 T^25,34^. The working group of Hore et al. optimized ^19^F photo-CIDNP experiments including ^19^F-labeled proteins. Kuprov et al. developed a very detailed theoretical analysis including relaxation and diffusion during the process of the hyperpolarization of 3-fluoro-tyrosine^17^. Our results show that ^19^F photo-CIDNP can be used also at magnetic fields of 0.6 T which is the typical range of benchtop NMR/MRI.

Although our experimental setup is optimized for imaging, the ability to acquire spectra from different nuclei simultaneously with a sufficient resolution proved to be very beneficial in optimizing signal acquisition. One can immediately check the signal strength of both nuclei as well as the SNR and the position of the resonance lines (Fig. 9, Fig. SI 2). The dual-core option also allows for the compensation for Larmor frequency fluctuations (due to residual minimal magnet temperature fluctuations) without the need to adding a reference substance. The prerequisite is that one of the substances of the system (solvent/chromophore/target molecule) exhibits a sufficient SNR in each individual measurement to serve for internal locking. Here, the ^1^H nucleus of the solvent (H_2_O) was used to post-process both the ^1^H and ^19^F spectra by correcting the phase drift prior to signal averaging. Thus, even non-illuminated ^19^F spectra could be acquired, which served as a reference for the determination of the mean SE (s. below). For more technical details see^16^.

The hyperpolarized spectrum could be further resolved by extending the acquisition time of the FID four-fold. The spectrum showed a doublet (Fig. 4c) in which the difference of the two resonance lines was 8.5 Hz, giving the J-coupling between the ^19^F and the vicinal proton of the aromatic ring system. However, this increased resolution was not used for the standard protocol because the increased noise contribution would have required more averaging, resulting in longer total acquisition time and higher total illumination time, which in turn would have resulted in faster bleaching of the chromophore. We also refrained from acquiring routinely non-irradiated spectra with a higher resolution, since the standard protocol already required several thousand averages in which additional chemical reactions occurred.

Accelerating the data acquisition for MRI required a trade-off between fast measurements with low spatial resolution and longer-duration data acquisitions needed to resolve the spatial distribution of the hyperpolarization in more detail, which is required for biomedical applications. For the mere detection and approximate localization of the substance, rapid acquisition of low-resolution images may be sufficient. These might also be used to monitor the dynamics of a target molecule in terms of both localization and metabolism^35^. However, increasing the resolution increases the time required for averaging. The time scale is also determined by substance-specific parameters such as relaxation times, sequence-specific parameters such as pulse angle, gradient duration, recording principles^36^, and the illumination time. The fast spin-echo MRI technique, widely used in routine medical MRI, also allowed to acquire a full low-resolution image within 4 s which was mainly due to the duration of the illumination time. The repetition time of 4 s is somewhat short compared to *T*_*1*_ and decreases the maximum achievable signal but was chosen to render the overall image acquisition not too long when acquiring many averages. However, in the future, we will also investigate whether faster sequences and shorter light exposure times with increased irradiation power can lead to acceptable images^36^.

Another limiting factor in fast spin-echo imaging is the *T*_*2*_ relaxation of the hyperpolarization, which determines the number of echoes that can be acquired with sufficient SNR. While smaller image matrices can be fully acquired after only one 90° pulse (e.g. Figure 3a), higher resolution usually requires segmented acquisition each preceded by a time intervall required for illumination in order to regenerate the hyperpolarization again. Figure 3b and c show the differences of recording all phase-encoded rows of the oversampled raw data matrices in the so-called k-space after one irradiation (Fig. 3b) compared to acquiring only a quarter of the raw data matrix before generating new hyperpolarization again (Fig. 3c). With a *T*_*2*_-relaxation of about 160 ms the signal was still high enough to acquire the full image in one shot (Fig. 3b) (please note that image encoding started at the center of the k-space). The SNR of the segmented image is about 50% higher than that of the non-segmented image (both having the same anumber of averages). However, this gain in SNR comes with an approximately four-fold longer acquisition and irradiation time. The latter ultimately leads to a faster bleaching of the sample.

The ordering of the phase-encoded rows in the k-space matrix prior to Fourier-transforming also influences the image contrast. Roughly speaking, the inner part of the k-space determines the brightness and the outer part determines the finer details of the image after Fourier transformation. Here we have chosen an order starting in the center of the k-space favoring overall SNR over image details. Finer details can be degraded due to the reduced signal when encoding the outer parts of k-space later in the echo train. This is seen by comparing Fig. 3b and c. In the latter figure, the central (*I*_*1*_) and peripheral regions (*I*_*2*_) appear better differentiated and exhibit a higher SNR than in Fig. 3b. In the former figure, the time for acquiring the image is almost twice of the *T*_*2*_ time. Thus, the signal has significantly decayed when acquiring the higher frequencies. This may lead to a blurring effect in phase-encoding direction which could lead to an apparently larger hyperpolarized volume. However, new technical solutions may be required as the standard strategy of increasing the SNR by increasing the number of averages and/or increasing the irradiation power is hampered by the inevitable chromophore bleaching and possible accumulation of by-products. We found that these effects became evident after several hundred irradiation cycles.

With the help of the complementary information from spectroscopy and imaging, the mean SE factor and the heterogeneous distribution of the hyperpolarization degree could be estimated. In most photo-CIDNP experiments, great efforts are made to ensure homogeneous illumination^37,38^, but studying biomedical samples will inevitably lead to an inhomogeneous distribution of the degree of hyperpolarization, since these samples are usually quite heterogeneous. The estimate of the mean SE should be currently seen as a first approximation, since we only reached about 80% of the maximum hyperpolarization as a compromise between irradiation duration and bleaching (Fig. SI 1). The estimated value of SE may also be smaller because the correction factor depends on the hyperpolarized volume which may be larger if including partial volume voxel. However, compared to the region depicted in Fig. 6b partial volume voxels were rather scattered and therefore excluded. *T*_*2*_-blurring may lead to overestimating the true distribution thus leading to a contrary effect. To account for these uncertainties that require more systematic experiments SE is estimated being in the order of 10^3^. Another factor was that the FMN concentration was much lower than the concentration of favipiravir. One molecule of the chromophore thus corresponded to about ten molecules of favipiravir. It can be speculated, that the number of favipiravir molecules could be significantly reduced before the hyperpolarized signal decreases significantly. Indications of this effect can actually be seen in Fig. SI 3. The SE per molecule could therefore be higher than estimated here.

However, the SE measured here still enabled the clear detection of hyperpolarized ^19^F even in high-resolution MRI data, with lower limits of < 500 pmol amounts of favipiravir per voxel. With the exception of a few spatially resolved photo-CIDNP studies^15,16,29^ signal amplification due to photo-CIDNP is usually reported from spectroscopic measurements with concentrations of the hyperpolarizable substance between 2 mM and 4 mM. Since the spectroscopic signal comes from the entire sample, efforts are made to illuminate the entire sample as uniformly as possible^37,38^.

Recent developments in LED-based *Low Concentration (LC)-photo-CIDNP* were in the range of 1 μM^39^ or lower. Yang et al.^24^ used 500 nM of the amino acid tryptophan in their fast-pulsing LED-enhanced NMR experiments. Three years later they reported the detection of only 20 nM tryptophan when acquiring an LED-irradiated enhanced signal of a proton in a side chain in tryptophan (Trp-α-^13^C-β,β,2,4,5,6,7-d_7_)^19^. Most of these studies have been performed with molecules containing aromatic systems with a hydroxy group such as tyrosine, or with tryptophan, which also contains nitrogen in a second ring system. Recently, Mompeán et al. reported sub-pmol detection sensitivity using a microcoil setup at 9.4 T to acquire a photo-CIDNP-enhanced ^19^F signal of 0.8 μM amount of p-fluorophenole (concentration 2.758 mM) in a detection volume of 1 μL^40^.

Compared to spectroscopic measurements, MRI measurements offer the opportunity to directly detecting regions with different degrees of hyperpolarization and thus potentially reducing the detection limit without special hardware. Lower degrees of hyperpolarization can either be due to lower amounts of substance or reduced light intensity, as seen in the peripheral parts of the light cone in our experiment. In comparison to a previous ^19^F hyperpolarized MRI study^16^ we investigated a heteronuclear system here. Favipiravir is a pyrazine carboxamide and contains two nitrogen atoms, and a hydroxy group is substituted to this heteroaromatic system. Compared to 3-fluoro-tyrosine previously studied by our group, the hydroxy group and the fluorine nuclei in favipiravir have a larger distance. In addition, there are fewer ^1^H and ^19^F couplings. Therefore, the hyperpolarization in favipiravir may be less distributed within the molecule and be more efficiently transferred to the ^19^F nucleus, which in turn may be one potential cause for the lower SE in 3-fluoro-tyrosine. Interestingly, the structural similarity of the hetero-aromatic ring in favipiravir to the middle part of riboflavin is immediately apparent^32^. Wörner et al. published photo-CIDNP measurements for non-classical disproportionation^41^. The effect of charge redistribution within the molecule is reflected in the hyperpolarizability of these systems. Further studies to analyze potential mechanisms will be necessary.

As further difference from our 3-fluoro-tyrosine study, we found that favipiravir changed much faster when left in the magnet at 303 K for several days (which is necessary to acquire several non-illuminated ^19^F spectra with sufficient SNR). While 3-fluoro-tyrosine remained stable for several days^16^, favipiravir showed an additional ^19^F signal in the non-illuminated spectra just after one day, while the hyperpolarized ^19^F spectra remained largely constant for the first four days (Fig. SI 3f). This additional ^19^F signal, which is shifted to lower frequencies by approximately 1 kHz (44 ppm) relative to the native ^19^F favipiravir signal (see SI) did not appear in the hyperpolarized spectra meaning that this substance might not be hyperpolarizable (Fig. SI 3).

**Figure 7:**
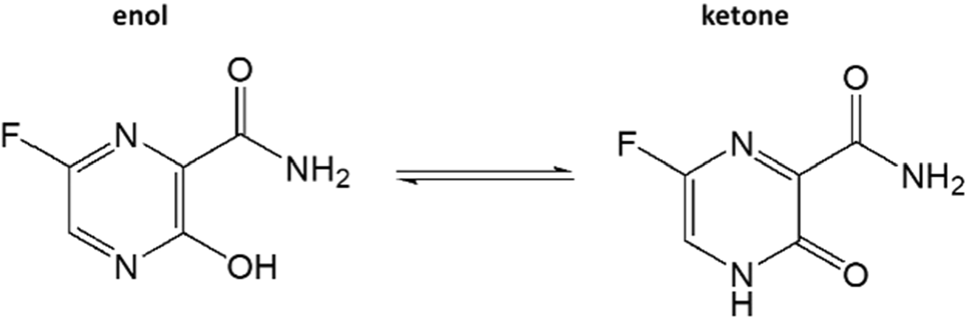
Enol and ketone tautomers of favipiravir. The ^19^F of the enol form used for the experiment was hyperpolarizable due to the N-heteroaromatic ring system and the OH group. The ^19^F of the ketone form showed no detectable hyperpolarization (Fig. SI 3).

Although for technical reasons we have not yet been able to precisely determine the molecular structure of the unknown substance, it might be assumed that the additional signal originates from one of the numerous isomeric structures of favipiravir. This molecule has two protonatable nitrogen nuclei in the ring system and the OH group, which can be converted into a ketone group by rearrangement (Fig. 7). It was shown that nine isomers can be formed under different conditions (pH values)^42^. Theoretical calculations of the transitions between the tautomeric forms were published by^43^. Due to the high sensitivity of the ^19^F nucleus to chemical shifts, any structural molecular change affects the position of the one fluorine signal. However, SE in the photo-CIDNP measurements are only expected for the N-heteroaromatic systems that have both a conjugated double bond system and a hydroxyl group. Thus, the detected signal, which was enhanced at neutral pH, can be unequivocally assigned to the enol form.

The results presented here show how new strategies may allow MR-based techniques to close the gap to other molecular imaging techniques. An interesting approach may be represented by combining photo-CIDNP MR spectroscopy with optical spectroscopy in the ps- and ns-time domain^44^, which allows the analysis of the rapidly occurring primary radical pair reactions. Future experiments will also include 3D imaging sequences to obtain full spatial information about the hyperpolarization distribution. However, this requires additional time-consuming optimization of the TSE sequence in terms of irradiation timing, excitation and refocusing pulses, segmented acquisition, etc., and was therefore outside the scope of this study.

Several limiting factors of this study have to be mentioned: (a) The effects of longer illumination with longer measurement schemes lead to chromophore bleaching, which ultimately prevents the sample from being optimally hyperpolarized and thereby limits the increase in spatial resolution. Potentially phototoxic side products can also limit biomedical applications^45^. (b) The results for the *T*_*1*_ and *T*_*2*_ measurements should be seen as preliminary. Here, more experiments are necessary to analyze potential dependencies on concentration of chromophores and target molecules, pH, temperature, and chromophore status (fresh vs. partially bleached). (c) A more unambiguous determination of the three-dimensional distribution of the hyperpolarization requires 3D measurements. However, 3D measurements are more time-consuming and require optimization of data acquisition or the use of other fast sequences^36^.

In summary, unlike other hyperpolarization techniques, photo-CIDNP offers the possibility to hyperpolarize fully biocompatible model systems. The experimental setup used here also provided additional information about the relaxation times of ^19^F hyperpolarization in favipiravir at 0.6 T. The same strategy may be used for other substances and other nuclei^38,46–50^ such as ^31^P, ^13^C, and ^15^N as well because appropriate coils are available. In particular, combining MRI and dual-core spectroscopy can provide new results about hyperpolarization transfer between different nuclei and may offer complementary results to standard high-field spectroscopy and MRI.

## Methods

**Figure 8:**
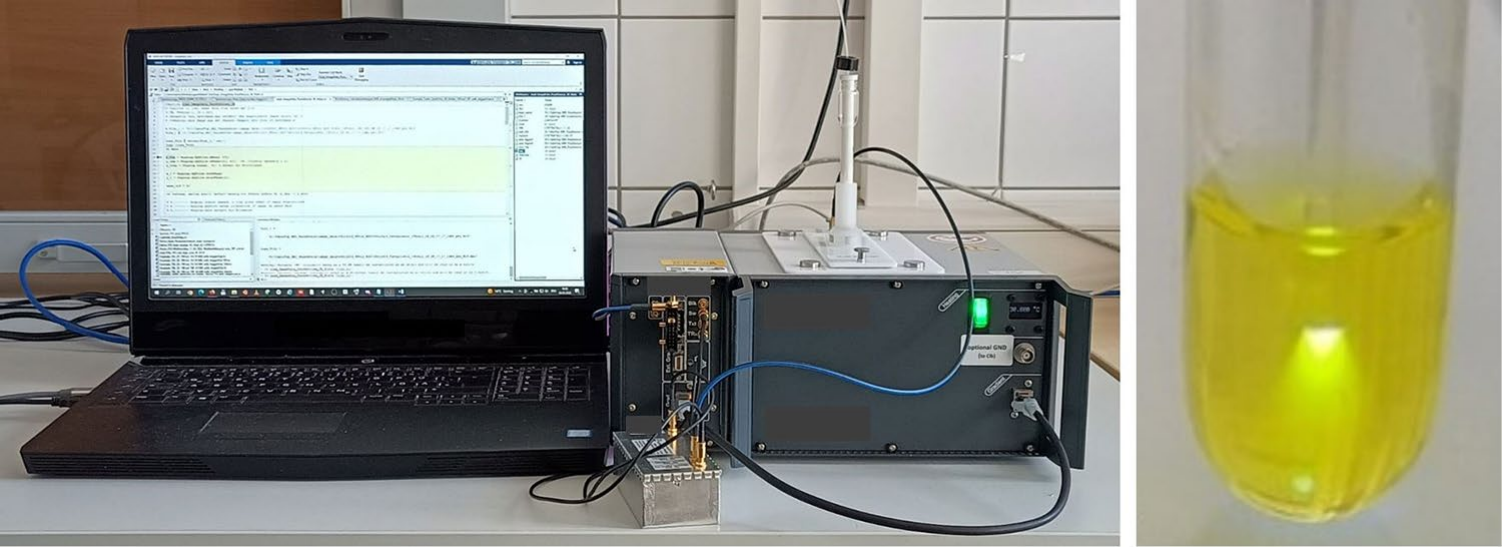
Experimental setup. *Left image*: Tabletop MRI with magnet, controller, small preamplifier and laptop. *Right image*: NMR tube with fiber positioned in pure H_2_O containing FMN and favipiravir.

Favipiravir (6-fluoro-3-hydroxypyrazine-2-carboxamide, c = 2.758 mM, (purchased from *abcr* GmbH, Germany) and riboflavin-5ʹ-monophosphate (FMN) sodium salt hydrate (c = 0.279 mM, purchased from Sigma Aldrich) were dissolved in 600 µL H_2_O_dest_ and filled into a 10 mm diameter NMR glass tube (Fig. 8). A low-cost high-power blue-light LED (CREE XP-E, high-power LED 455 nm) was used for photoexcitation of riboflavin. The light was coupled into an optical fiber (THORLABS, Ø1000 μm Core Multimode Fiber, Low OH). The light intensity, measured at the tip of the fiber, was typically 6 mW. For positioning of the glass fiber, a home-made 3D-printed cylinder with a central hole of about 1 mm was inserted into the glass tube (Fig. 8).

^19^F and ^1^H spectroscopic and imaging experiments were performed with a tabletop low-field permanent magnet (0.58 T, Pure Devices GmbH, Wuerzburg, Germany) using a broadband ^19^F coil that allowed the detection of both ^1^H and ^19^F signals. The setup was controlled by a MATLAB-based software environment provided by the vendor. Compared to our previous study^16^ we improved the SNR by an approximate factor of 7 by inserting a broadband preamplifier with approximately 22 dB amplification (Pure Devices GmbH, Wuerzburg, Germany). Data acquisition and technical details are described in more detail in^16^.

**Figure 9:**
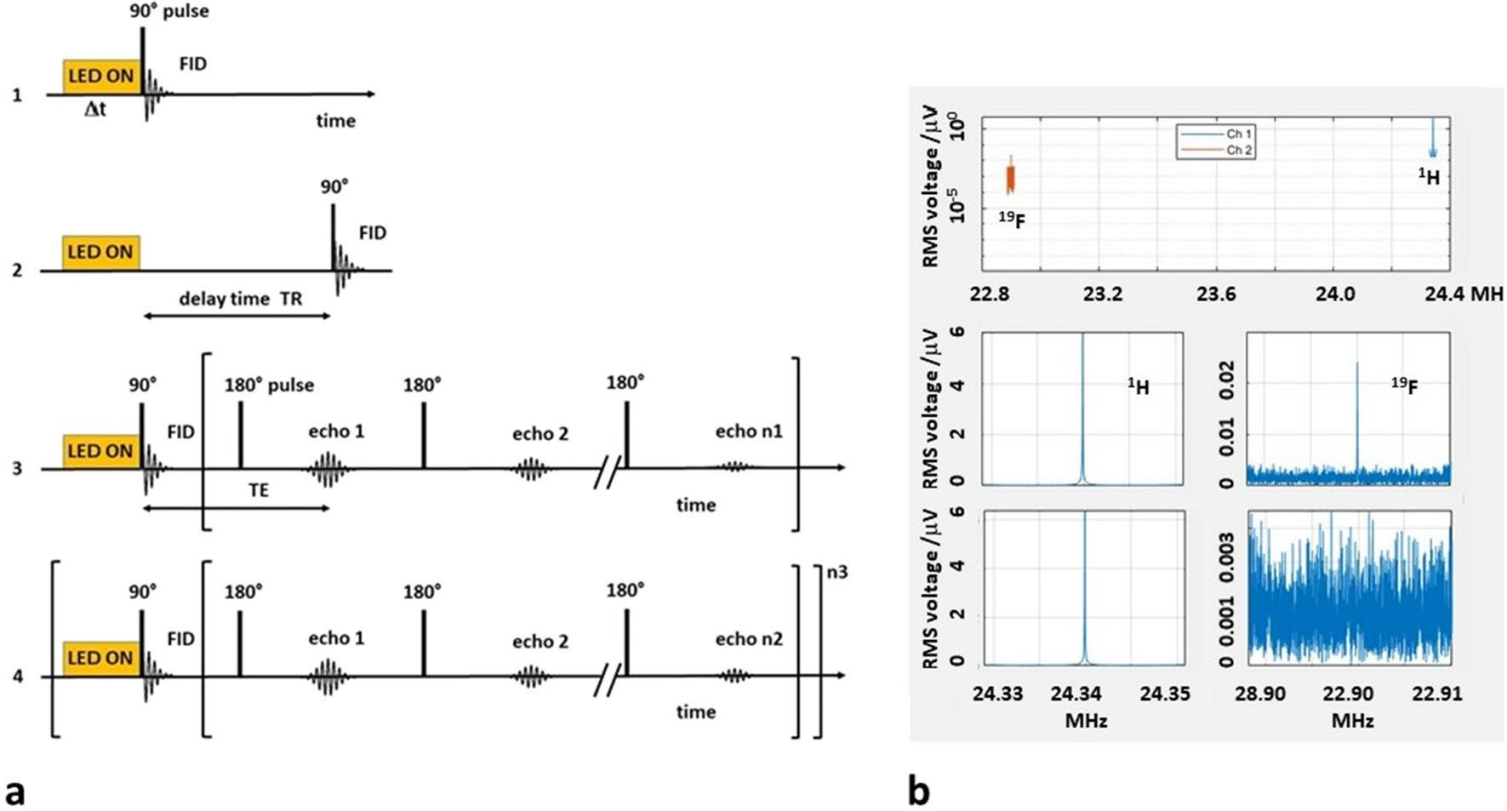
Photo-CIDNP MRI and dual core spectroscopy. (**a**) Data acquisition schemes. **(1)** Spectroscopy: the sample is illuminated for Δt seconds and the free induction decay (FID) is acquired immediately. **(2)** Scheme to determine *T*_*1*_-relaxation using a variable delay time between illumination and FID acquisition. **(3)** TSE MRI: n1 phase-encoded echoes (i.e. rows of the raw data matrix) are acquired after one 90° pulse preceded by one illumination block of Δt seconds duration. **(4)** Segmented TSE MRI: each of the n3 acquired echo trains fills n2 rows in the raw data matrix in the k-space. Filling the complete matrix requires n3 new excitation pulses and illumination blocks until the entire data matrix is filled. Each echo decays with *T*_2_* while the envelope of the echo amplitudes declines with *T*_*2*_. Therefore, *T*_*2*_ of the signal must be long enough to allow to acquire n1 respectively n2 echoes after each excitation with sufficient SNR. (**b**) Dual core spectroscopy (one data acquisition). *Upper subpanel*: signal after illumination (red: ^19^F signal, blue: ^1^H signal, y-axis plotted logarithmically). *Left lower subpanels*: ^1^H channel (with and without illumination). *Right lower subpanels*: ^19^F channel. After 15 s illumination prior to data acquisition, the ^19^F signal (*upper panel*) is clearly recognizable while without illumination the SNR was too low to detect the ^19^F signal (*lower panel*).

A 2D fast TSE sequence was used both for ^1^H and ^19^F MRI^51^. Unless otherwise specified, a gauss-shaped pulse (duration about 40 μs) was used for the 90° excitation RF pulse, with TE = 4 ms, TR = 4 s, and a four-fold read- and phase-encoding oversampling. With TSE methods^36,51^, several k-space rows with varying phase-encoding are acquired after the irradiation of a 90° pulse (TF indicates the corresponding number of rows, Fig. 9). TF could be varied from the total number of phase-encoded k-space rows (including phase-oversampling) to smaller values. In the latter case, several 90° pulses and illumination blocks are necessary prior to the acquisition of each echo train with a subset of rows in the k-space matrix. This may be necessary if the lifetime of the hyperpolarization is less than the time required to acquire all k-space rows at once. Duration and onset of the illumination was triggered from the MATLAB-based sequence. Data were usually four-fold oversampled in phase- and in read-encoding direction, which resulted in a four-times-four-fold increased raw data matrix. The oversampling in phase-encoding direction accordingly prolonged the acquisition time. Phase-encoding started from the center of the raw data matrix, i.e., at the center of the k-space.

The amplitudes of the ^1^H signals remain rather constant, regardless of whether the sample is illuminated or not, since the ^1^H signals originate almost entirely from the solvent (H_2_O). The ^1^H signal can therefore be used for internal correction of magnetic field fluctuations before averaging multiple acquisitions. In the displayed measurement, the amplitude of the hyperpolarized ^19^F signal was approximately 4 × 10^–3^ of amplitude of the ^1^H signal (note that the coil sensitivity is only approximately 15% at ^1^H Larmor frequency). For further details see^16^.

For spectroscopic experiments, multiple nuclei could be acquired either individually or simultaneously. A composite 90° pulse was used for excitation of both the ^1^H and the ^19^F nuclei. The duration of both the ^1^H and the ^19^F 90° pulse component was approximately 450 μs but the driving amplitude of the ^19^F pulse was only approximately 15% that of the ^1^H pulse due to the reduced coil efficiency at the ^1^H Larmor frequency. The ^19^F Larmor frequency for favipiravir was shifted by −10.5 ppm (−240.45 Hz) with respect to the default ^19^F Larmor frequency of about 22,901,443 Hz.

For long-term acquisition of up to several thousand free induction decay (FID) signals, data were post-processed prior to averaging. This accounted for minimal fluctuations in the B_0_ field due to remaining temperature fluctuations of the magnet (kept at 303 K) despite the precise temperature control. The corresponding MATLAB routine was provided and optimized by Pure Devices GmbH. For further details of the experimental procedures see^16^.

The relaxation times were determined after a hardware upgrade (gradient amplifier DC-600, HF pulse amplifier RF-100, Pure Device GmbH, Würzburg, Germany). Furthermore, the coupling of the LED into the fiber optic was improved, resulting in a typical output power of 16 ± 2 mW. The concentration of favipiravir (2.546 mmol/L) was slightly lower than in previous measurements. All other parameters remained unchanged. To determine *T*_*2*_ from hyperpolarized ^19^F, we modified a standard multi-echo sequence based on a CPMG acquisition scheme to allow averaging of the weak echo signals from ^19^F using a simultaneously acquired ^1^H signal.

The absolute values of the ^19^F signal maxima were fitted to a single exponential function using OriginLab 2023b (https://www.originlab.com/origin). The lifetime (*T*_*1*_) was determined similarly to Sheberstov et al.^38^ by using a fixed illumination time (here: 6 s) and varying the delay time before the FID was detected (Fig. 9a2). Four measurements were averaged for each delay time, except for the two longest delay times (8 s and 12 s), where eight measurements were averaged due to the lower SNR. Absolute values of the ^19^F signal maxima were fitted with a single exponential function using OriginLab 2023b.

### Supplementary Information

Supplementary Information.

## Data Availability

The data are available on request from the corresponding author.
