# Supplementary material for: Detection of sub-nmol amounts of the antiviral drug favipiravir in 19F MRI using photo-chemically induced dynamic nuclear polarization
Source: Sci Rep. 2024 Jan 17;14:1527. doi: 10.1038/s41598-024-51454-4 (PMC10794400; doi:10.1038/s41598-024-51454-4)
Supplement: Supplementary file 1 — Supplementary Information. [file 41598_2024_51454_MOESM1_ESM.pdf]

Supplementary information

Detection of sub-nmol amounts of the antiviral drug favipiravir in ^{19}F MRI using photo-chemically induced dynamic nuclear polarization.

J. Bernarding[1*], C. Bruns[1], I. Prediger[1], M. Mützel[2], M. Plaumann[1]

Affiliations

[1]Institute for Biometry and Medical Informatics, Otto-von-Guericke University Magdeburg, Leipziger Strasse 44, D-39120 Magdeburg, Germany

[2]Pure Devices GmbH, D-97222 Rimpar, Germany

[*]Corresponding author: Johannes Bernarding, johannes.bernarding@med.ovgu.de

1. Signal enhancement as a function of illumination time

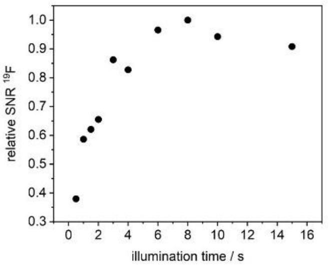

Figure SI 1. SNR (relative to the maximum) of photo-CIDNP-hyperpolarized ^{19}F signal of favipiravir as a function of illumination time. Four measurements were averaged for each illumination time. Delay between consecutive data acquisitions 11 s. Phase- corrected real values of the spectra were integrated and normalized to the values of the integrated ^{1}H spectrum. An illumination time of 4 s corresponded to approximately 80% of the maximum achievable signal enhancement.

2. T_2* times of ^{1}H and ^{19}F

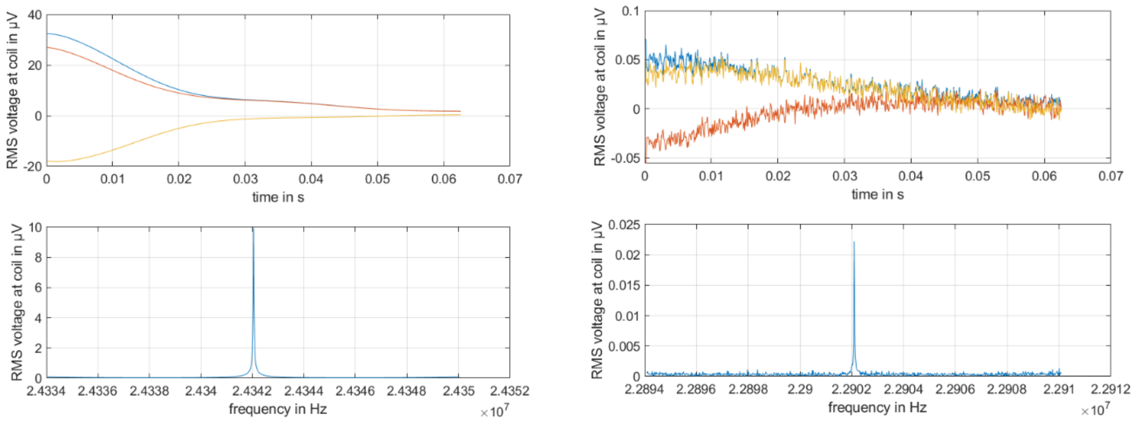

Figure SI 2. FIDs of ^{1}H (left, upper panel) and simultaneously acquired photo-CIDNP-hyperpolarized ^{19}F (right, upper panel) spectra. Illumination time was 2 s, eight measurements were averaged (blue: absolute value, red: real part, yellow: imaginary part).

The ^{1}H FID representing almost entirely the solvent signal is recorded over the whole sample leading to a non-expontial decay due the increased B_0-field inhomogeneity. The ^{19}F FID stems from a much smaller field-of-view exhibiting a more homogeneous B_0-field. T_2* for ^{1}H was estimated by the vendor-provided shim-sequence to about 32 ms which is within the same range as T_2* of the ^{19}F signal. The lower panels show the according resonance lines.

3. Spectroscopy with and without illumination

Fig. SI 3 shows spectroscopic measurements with and without illumination. Because of the low concentration of favipiravir non-illuminated measurements required hours to days. Each data acquisition of a non-illuminated spectrum was preceded by the acquisition of at least one hyperpolarized spectrum (32 averages) to check the integrity of the system (illumination duration 4 sec). For non-illuminated spectra, measurement times of up to 32 h were required. Bandwidth was 1 kHz or 4 kHz, sampling rate 16k, TR 10 s (including 4 s illumination time for measurements of hyperpolarized spectra).

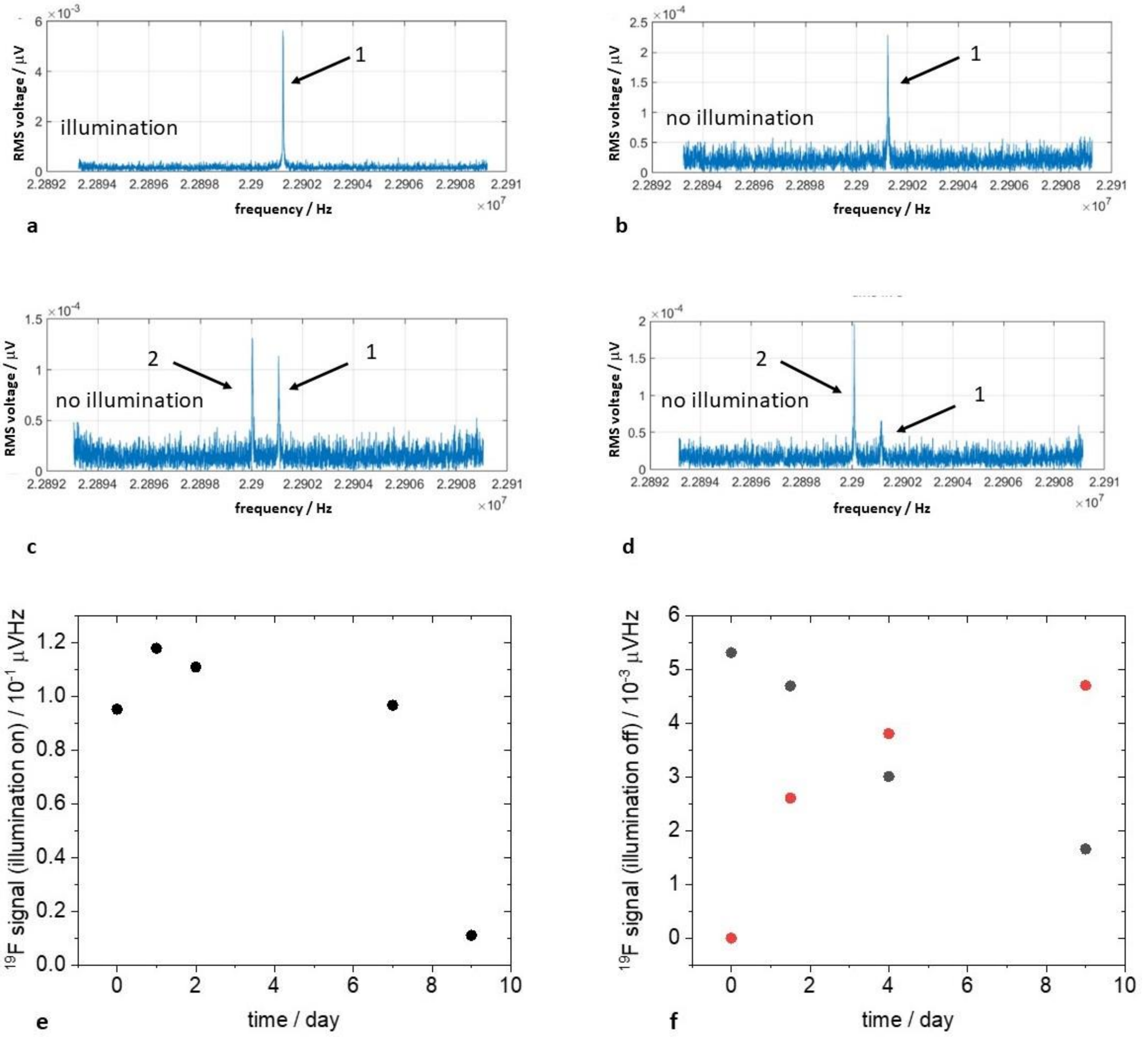

Figure SI 3. Long-term time measurements of the ^{19}F signal in favipiravir.
a-d: Absolute values of selected spectra (complete bandwidth) show the hyperpolarized ^{19}F signal (1 in Figure SI 3a) in the illuminated sample. The corresponding ^{19}F signal (1) in the non-illuminated sample is slowly decreasing while an additional increasing second signal (2) is seen. Signal 2 is not present in the spectrum of the illuminated sample (a).
a: Illuminated sample at day 0 (32 averages). **b:** Non-illuminated sample at day 0 (2048 averages). **c:** Non-illuminated sample at day 4 (4096 averages). **d:** Non-illuminated sample at day 9 (3553 averages).
^{19}F signals were re-calibrated to the first measurement under illumination with respect to bandwidth and signal fluctuations using the ^{1}H signal, which were due to remaining minimal temperature fluctuations in the temperature-stabilized magnet.
e, f: Integrated phase-corrected real values of the ^{19}F spectra for illuminated samples (e) and non-illuminated samples (f) as a function of time. Black dots correspond to signal 1and red dots to signal 2.

Interestingly, the spectra of the non-illuminated samples showed a second signal, which was detected already after day one. This signal was shifted to lower frequencies by approximately 44 ppm (1000 Hz) and was not seen in spectra under illumination. The amplitude of this second signal (signal 2 in Figure SI 3c-d) increased with time (Figure SI 3f), whereas the original favipiravir signal decreased (signal 1 in Figure SI 3b-d, Figure SI 3f). In

contrast, the hyperpolarized signal changed little until day 7. Only from day 9 on, the sample showed a strong decrease of the hyperpolarized signal (Figure 3e).

Thus, unlike 3-fluorotryrosine [1], the hyperpolarizable form of favipiravir appears to be less stable when stored in the magnet at 303 K, even with little to moderate illumination (hyperpolarized spectra were recorded between the non-illuminated spectra). The only slightly varying degree of the hyperpolarization between day 0 and day 7 suggests a relatively constant amount of hyperpolarizable favipiravir. We have not yet determined the molecular structure of the second molecule because of the necessary difficult and lengthy experiments, but a slow transition of the original hyperpolarizable enol form of favipiravir [2] to the ketone form may provide an explanation [3] (see main text discussion section).

The observation that the signal 2 increased while the hyperpolarized signal remained relatively constant may be explained by the lower riboflavin concentration compared with the favipiravir concentration: sufficiently hyperpolarizable favipiravir molecules remained even when the concentration of the enol form was greatly reduced (day 4-7). Further experiments, such as changing the pH, are needed to gain additional insight into possible mechanisms of the transition between enol and ketone forms. For the ease of the reader, figure 7 of the main text is shown here as figure SI 4.

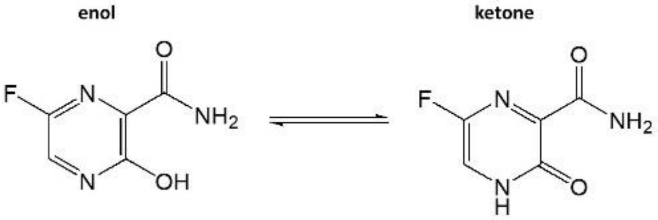

Figure SI 4: The ^{19}F of the enol form is hyperpolarizable due to the heteroaromatic ring system and the OH group. In the ketone form, the ring system has undergone a transition and the H nucleus is now bound to the N prohibiting the transfer of the hyperpolarization to the ^{19}F. Therefore, no ^{19}F hyperpolarization could be detected in the ketone form.

References

1. Bernarding, J., Bruns, C., Prediger, I. & Plaumann, M. LED-Based Photo-CIDNP Hyperpolarization Enables ^{19}F MR Imaging and ^{19}F NMR Spectroscopy of 3-Fluoro-DL-tyrosine at 0.6 T. *Appl Magn Reson* **53,** 1375–1398 (2022).
2. Furuta, Y. *et al.* T-705 (favipiravir) and related compounds: Novel broad-spectrum inhibitors of RNA viral infections. *Antiviral research* **82,** 95–102 (2009).
3. Antonov, L. Favipiravir tautomerism: a theoretical insight. *Theoretical chemistry accounts* **139,** 145 (2020).
